# A New Case of Prenatally Diagnosed Pentasomy X: Review of the Literature

**DOI:** 10.1155/2015/935202

**Published:** 2015-01-29

**Authors:** Linda Maria Azzurra Pirollo, Leila Baghernajad Salehi, Simona Sarta, Marco Cassone, Maria Vittoria Capogna, Emilio Piccione, Giuseppe Novelli, Adalgisa Pietropolli

**Affiliations:** ^1^Section of Gynecology and Obstetrics, Academic Department of Biomedicine and Prevention and Clinical Department of Surgery, Tor Vergata University Hospital, Viale Oxford 81, 00133 Rome, Italy; ^2^Laboratory of Medical Genetics, Polyclinic of Tor Vergata Foundation, Viale Oxford 81, 00133 Rome, Italy; ^3^Laboratory of Medical Genetics, University of Rome Tor Vergata, Via Montpellier 1, 00133 Rome, Italy; ^4^Genetics Section, Department of Biomedicine and Prevention, University of Rome Tor Vergata, Via Montpellier 1, 00133 Rome, Italy

## Abstract

Pentasomy X is a rare chromosomal abnormality probably due to a nondisjunction during the meiosis. Only four cases prenatally diagnosed were described until now. Our case is the fifth one prenatally diagnosed at 20 weeks of gestational age in a 39-years-old woman. She underwent invasive prenatal diagnosis for her advanced maternal age without any other known risk factor. Amniocentesis performed at 17 weeks showed a female 49, XXXXX karyotype. The ultrasonographic examination revealed nonspecific signs of a mild early fetal growth retardation and no significant increased nuchal fold. The fetal autopsy and the X-ray excluded major malformations. Prenatal diagnosis is often difficult due to the lack of indicative ultrasonographic findings and the rarity of described cases. The influence of the mother's age on the occurrence of penta-X syndrome has not been determined. Considering the lack of correlation between advanced maternal age and increased risk for pentasomy X, as well as the absence of typical echographic signs, evaluation of the inclusion of a noninvasive prenatal test (NIPT) that expands clinical coverage to include the X and Y chromosomes in routine prenatal diagnosis should be considered as well as three-dimensional ultrasound to detect any helpful indicative prognostic signs.

## 1. Introduction

Pentasomy X (49, XXXXX) is a very rare aneuploidy involving sex chromosome X. It is characterized by a variable phenotype in females.

The described features in 49, XXXXX karyotype include severe mental retardation with delayed speech development, short stature, facial dysmorphisms, osseous and articular abnormalities, congenital heart defects, and skeletal and limb abnormalities [1]. The craniofacial anomalies may include microcephaly, micrognathia, plagiocephaly, hypertelorism, upslanting palpebral fissures, a flat nasal bridge, and ear malformations. The hands and feet are generally small and camptodactyly, clinodactyly, and radioulnar synostosis are common findings. Immunoglobulin anomalies and an increased susceptibility to infection have also been reported.

The incidence of pentasomy X is unknown because of rarity of this aneuploidy but some authors indicate about 1/85000 comparing to 49, XXXXY in males [2].

The pathogenesis of pentasomy X is not clear: this aneuploidy must arise as a result of a meiotic malfunction, either maternal or combined maternal and paternal in origin [3–5].

Only four pentasomy X cases were detected prenatally and the ultrasonographic findings are summarized in Table 1 [6–9].

## 2. Case Presentation

**Figure 1:**
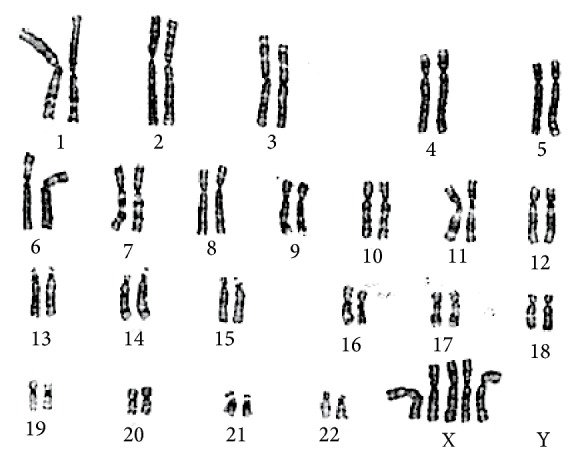
Fetal karyotype demonstrating pentasomy X.

**Figure 2:**
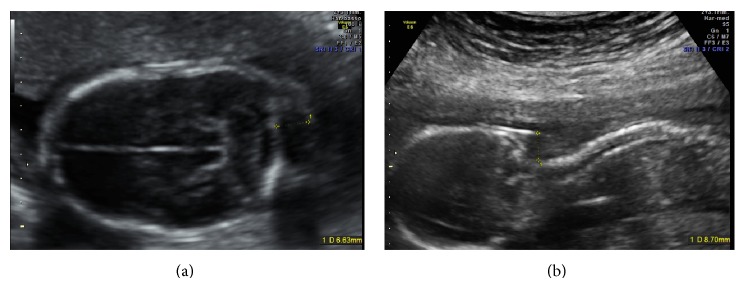
Increased nuchal fold in a transverse view (a) and in a longitudinal view (b) of the fetal head. The maximum value obtained was 8.7 mm. Such subcutaneous oedema was not described in the autopsy report.

**Figure 3:**
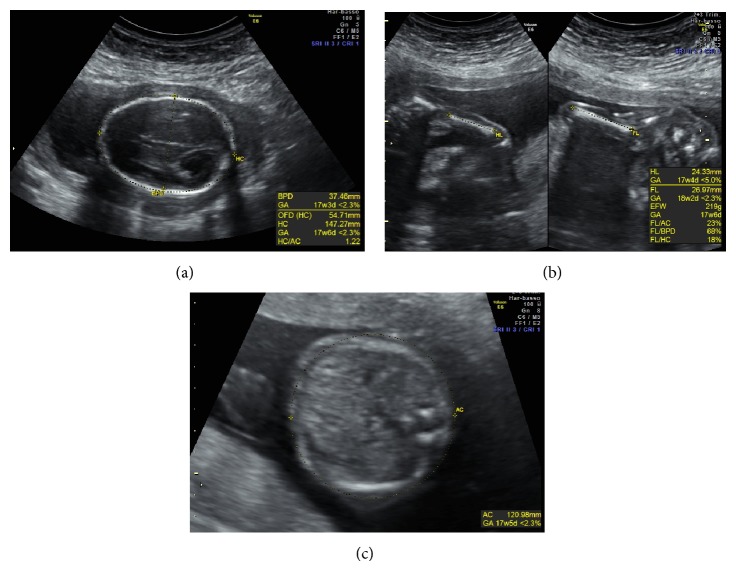
Fetal biometry: biparietal diameter, frontooccipital diameter, and head circumference (a), humerus and femur length (b), and abdominal circumference (c). The parameters resulted under the 5° pc of the standard for the gestational age (20 weeks of pregnancy).

**Figure 4:**
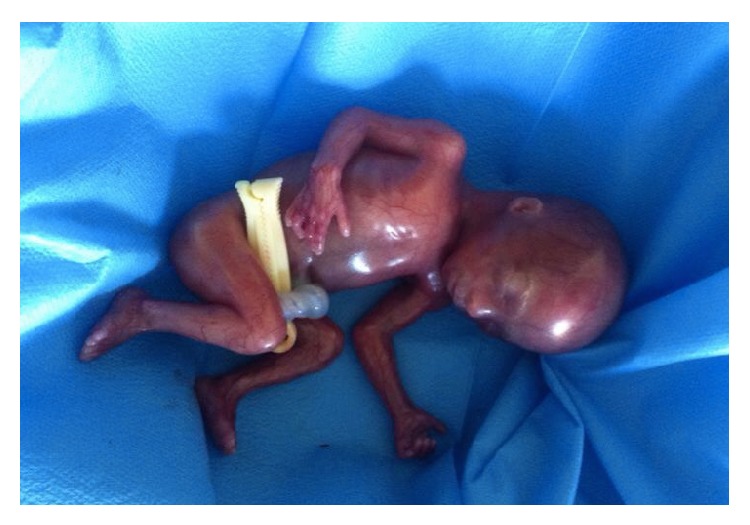
Fetal autopsy: it confirmed the absence of major malformations.

A 39-year-old healthy woman referred to the Prenatal Diagnosis Centre of Tor Vergata University Hospital, Rome, to undergo genetic amniocentesis at 17 weeks of pregnancy. The main indication to the procedure was advanced maternal age. She had a noncontributive history (4G2P) with a previous spontaneous miscarriage at 10 weeks between the two pregnancies. She was smoker (8 cig./day) with a weight of 75 kg (BMI 29.3). Before the procedure, the patient was evaluated with some blood tests (blood group, indirect Coombs test, HbsAG, HCV, HIV, VDRL, and TPHA), ultrasound exam, and vaginal swabs in order to predict a possible ascendant infection. Antibiotic prophylaxis (Azithromycin (Zithromax)) was administered as specific dose together with vaginal progesterone for six days prior to the procedure. Written informed consent was obtained. Amniocentesis was performed and the amniotic fluid sample was referred to the medical genetics department. Chromosomal analysis was performed in long-term amniotic fluid cultures from three separate tissue culture flasks. GTG- and CBG-banded metaphases were analysed and the karyotype was reported as 49, XXXXX (Figure 1). The family was concerned with the results and, during the counselling, they were offered an ultrasound scan before the mother's decision. At 20 weeks of gestational age the ultrasonographic examination ruled out major fetal malformation. As minor finding there was a mild nuchal fold increase and a fetal growth at lower values than would be standard for the gestational age (Figures 2 and 3). The woman decided to terminate the pregnancy: the fetal X-ray scan and autopsy excluded significant morphological alterations (Figure 4). No subcutaneous oedema was described in the autopsy report; this finding evaluated prenatally could be transient as it was previously hypothesized [9].

## 3. Discussion

Pentasomy X is a rare aneuploidy with variable phenotype. A review of postnatal pentasomy X cases (26 cases) shows mental retardation and development retardation to be the only clinical aspects in common in all the cases reported in literature (Table 2, [10–32]).

It seems depending upon an X gene dosage defect due to an alteration of X inactivation mechanisms. It is also noteworthy that the female triple X shows normal phenotype.

The rarity of the condition and the few data available make genetic counselling complicated in order to give detailed clinical information to the patient about the disorder; so a multidisciplinary counselling is requested involving obstetrical-gynecological evaluation to identify echographic patterns.

According to the reported postnatal pentasomy X cases, in which mental and development retardation were described as the constant relevant signs, genetic counselling was conducted.

Our case shows how prenatal counselling could be difficult: the absence of indicative echographic patterns for chromosomal abnormalities limits the definition of prognosis at birth.

**Figure 5:**
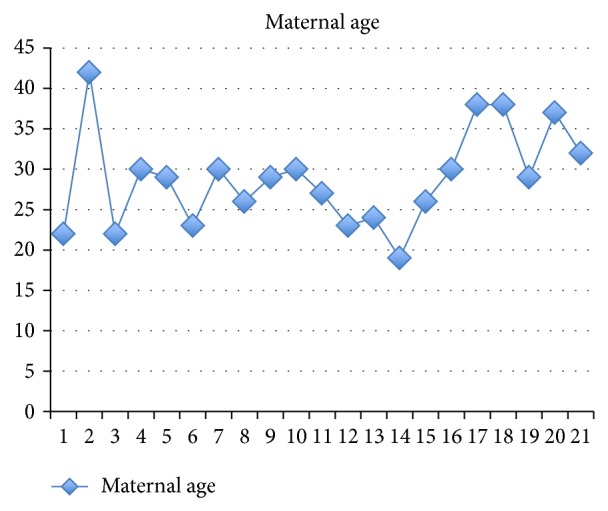
Distribution of maternal age in the 21 cases reported in literature [10–32].

Our patient required genetic counselling only on the base of her advanced maternal age. The influence of the mother's age on the occurrence of penta-X syndrome has not been determined so it could not be considered as risk factor (Table 3 and Figure 5).

It remains difficult to plan obstetric and gynecological counselling to identify which cases require genetic counselling and fetal karyotype.

At the ultrasound scan we observed an early, symmetric restriction of fetal growth and an increased nuchal fold: such features represent a weak reason for requesting a fetal karyotype. As the literature's review demonstrated most of the major malformations have a late onset so prenatal diagnosis is complicated.

In conclusion, our case emphasised the difficulty of medical practice due to the rarity of the condition, the absence of correlation with advanced maternal age, and the lack of representative ultrasonographic findings.

An early restriction of the fetal growth and an increased nuchal fold associated with an advanced maternal age could suggest referral to a fetal karyotype for further investigation. Fetal autopsy and X-ray represent an essential step for a correct grading of chromosomal abnormalities.

Considering that there is no correlation between advanced maternal age and increased risk for pentasomy X and the absence of typical echographic signs of this syndrome, the need to include a noninvasive prenatal test (NIPT), which expands clinical coverage to include the X and Y chromosomes in routinary prenatal diagnosis as molecular noninvasive tool, and three-dimensional ultrasound to detect any helpful indicative echographic prognostic signs should be evaluated [33].

## 4. Take Home Message

We conclude the following:

- no maternal age correlation (from literature);
- mental and development retardation always present;
- clinical phenotype due to X chromosome dosage defect;
- controversial clinical phenotype among X chromosome aneuploidies 47, XXX versus 49, XXXXX;
- NIPT as routine noninvasive tool screening for young pregnant female.

## Figures and Tables

**Table 1:** Review of the literature: cases prenatally diagnosed.

Case report	Maternal age	Ultrasonographic findings	Invasive prenatal test	Weeks of pregnancy	Autopsy report and fetal X-ray scan
Martini et al. 1993 [6]	39 years	(1) Growth restriction (2) Radioulnar synostosis	Amniocentesis performed after US scan	18 weeks	Hypertelorism, slight mongoloid slant, radioulnar synostosis, and hypoplastic ovaries depleted of oocytes

Myles et al. 1995 [7]	26 years	(1) Dandy-Walker malformation (2) Hydrocephaly (3) Ventricular septal defect (4) Hypertelorism (5) Polyhydramnios (6) Growth restriction	Amniocentesis performed after US scan	33 weeks	NO (born at 39 weeks with caesarean section and died at 134 days of age)

Cheng et al. 2008 [8]	29 years	Increased nuchal translucency	Chorionic villous sampling before US scan	11 weeks	No

Aytac et al. 2012 [9]	26 years	(1) Increased nuchal fold (2) Pleural effusion (3) Subcutaneous edema (4) Ascites (5) Bilateral hand clinodactyly	Amniocentesis performed after US scan	17 weeks	No

**Table 2:** Postnatal cases and clinical features: review of the literature.

Clinical signs	%	Number of cases	Reference
Facial anomalies	46	12	[10, 12, 14, 17–19, 21–23, 25, 28, 29]
Mental retardation	100	24^*^	[10–32]
Developmental retardation	100	26	[10–32]
Skeletal abnormalities	61	16	[10–13, 15–19, 22, 23, 29–31]
Craniofacial anomalies	46	12	[11, 13, 15, 16, 18, 28, 30–32]
Cardiovascular anomalies	58	15	[10–12, 14, 15, 18, 21, 23, 25, 28, 30, 31]

^*^Two cases were newborns and mental retardation was impossible to evaluate.

**Table 3:** Maternal age at conception when reported [10–32].

Maternal age	% (21 cases)
<35 years	81
>35 years	19
